# Spatiotemporal consistency of neural responses to repeatedly presented video stimuli accounts for population preferences

**DOI:** 10.1038/s41598-023-31751-0

**Published:** 2023-04-04

**Authors:** Ayaka Hoshi, Yuya Hirayama, Fumihiro Saito, Tatsuji Ishiguro, Hiromichi Suetani, Keiichi Kitajo

**Affiliations:** 1 RIKEN Center for Brain Science, 2-1 Hirosawa, Wako, Saitama 351-0198 Japan; 2 KIRIN Central Research Institute, Research & Development Division, Kirin Holdings Company, Limited, 26-1-12-12 Muraoka-Higashi 2-chome, Fujisawa, Kanagawa 251-8555 Japan; 3 Faculty of Science and Technology, Oita University, 700 Dannoharu, Oita, 870-1192 Japan; 4 Division of Neural Dynamics, Department of System Neuroscience, National Institute for Physiological Sciences, National Institutes of Natural Sciences, 38 Nishigonaka, Myodaiji, Okazaki, Aichi 444-8585 Japan; 5 Department of Physiological Sciences, School of Life Science, The Graduate University for Advanced Studies (SOKENDAI), 38 Nishigonaka, Myodaiji, Okazaki, 444-8585 Japan

**Keywords:** Neuroscience, Psychology

## Abstract

Population preferences for video advertisements vary across short video clips. What underlies these differences? Repeatedly watching a video clip may produce a consistent spatiotemporal pattern of neural activity that is dependent on the individual and the stimulus. Moreover, such consistency may be associated with the degree of engagement and memory of individual viewers. Since the population preferences are associated with the engagement and memory of the individual viewers, the consistency observed in a smaller group of viewers can be a predictor of population preferences. To test the hypothesis, we measured the degree of inter-trial consistency in participants’ electroencephalographic (EEG) responses to repeatedly presented television commercials. We observed consistency in the neural activity patterns across repetitive views and found that the similarity in the spatiotemporal patterns of neural responses while viewing popular television commercials predicts population preferences obtained from a large audience. Moreover, a regression model that used two datasets, including two separate groups of participants viewing different stimulus sets, showed good predictive performance in a leave-one-out cross-validation. These findings suggest that universal spatiotemporal patterns in EEG responses can account for population-level human behaviours.

## Introduction

In traditional mass marketing for a product or service awareness, companies invest enormous amounts of money in advertisements and repeatedly advertise via short videos on television (TV) and video-sharing sites such as YouTube. Although these are watched repeatedly under the same conditions, some videos are popular, eliciting the engagement and memory of individual viewers and inducing population responses involving preference, while others are considered boring. What kind of perception processes are related to the difference?

An increasing number of electroencephalography (EEG) and magnetoencephalography (MEG) studies use naturalistic audiovisual stimuli to investigate perceptual and cognitive processes^1–7^. Recent works have documented a growing link between certain features of such audiovisual stimuli and the consistency of neural responses, defined here as the reproducibility of EEG response waveforms in individuals across repetitive trials. For instance, naturalistic audiovisual stimuli elicit a prominent consistency of neural responses across repetitive trials^2,5^. The consistency of neural activity between the first and second views of short film clips often corresponds to arousing moments that induce viewer engagement, such as scenes marked by a high level of suspense, tension, or surprise^2^. Furthermore, scalp EEG responses to salient, noisy, time-varied visual inputs, which draw viewers’ attention, show a signature of consistency^8^. Thus, the EEG-level neural consistency observed across repetitive trials could be a robust phenomenon associated with viewers’ attention and engagement.

From the viewpoint of neuromarketing studies, it is an intriguing question whether we can predict larger population responses or behaviours from neural data obtained from a smaller sample of viewers. Prior studies aimed at predicting population responses or behaviours from brain activity measured with blood oxygen level-dependent signals^9–11^. With these approaches, the strength of group-averaged neural responses from a small sample of viewers is presumed to correlate with measures of population responses or behaviours, such as the purchasing decisions^10^ or the effects of the media^11^ in a large population. A few studies have applied EEG methodology to predict population responses or behaviours using neural data from a small sample of viewers. For example, three frequency-based frontocentral EEG measures (i.e. alpha/beta asymmetry, alpha/theta power and theta/gamma power) and the composite EEG score, which is an average of power from three frequency bands for every minute, were found to correlate with behavioural measures of minute-to-minute time series data for TV ratings and Twitter volume^12^. In addition, the inter-individual correlation of EEG activity in single trials was shown to be associated with the engagement of viewers and population responses^13,14^.

Despite the importance of repetitive viewings of video stimuli in our daily life, few studies investigated the relationship between the consistency of neural responses across repetitive viewings and population preferences. To our knowledge, the relationship between the consistency of neural responses across repetitive trials within viewers, which should be associated with the engagement and memory of individual viewers, and population preferences remains largely unknown.

We hypothesised that neural consistency of responses across repetitive trials from a small sample would be predictive of the preferences of a large population. We speculate that repeated watching of short videos alters individual brain states reflecting engagement and memory. Thus, the stimulus preferred by a large population (i.e. one that is popular) should maintain these individual brain states, observed as highly consistent spatiotemporal patterns of brain activity across trials. To test the hypothesis, we computed the spatiotemporal consistency of EEG recordings in response to repeatedly viewed TV commercials, and examined the link between consistency and population preferences.

## Results

### The relationship between the consistency of brain responses across repetitive views and population preferences

We assessed the consistency of brain responses across repetitive views of TV commercials within each participant. To assess the degree of consistency of individual brain responses across repetitive views (i.e. from the 1st to 10th trials), a canonical correlation analysis (CCA)-based method, which extracts correlated time-series components across repetitive trials, was first applied between the trial-pairwise EEG data across ten views in a round-robin design (i.e. for all possible combinations: _10_C_2_ = 45 combinations) within each participant and each TV commercial. We then extracted linear combinations of EEG signals with the eigenvectors as coefficients for canonical variates (i.e. the first three correlation-maximised components) to analyse the relationship between the degree of consistency and the population preference. The population preference index of all TV commercials was assessed as the total numbers of unaided recalls for a favourite TV commercial gathered from 3,000 people every month (see “Methods” for details).

**Figure 1:**
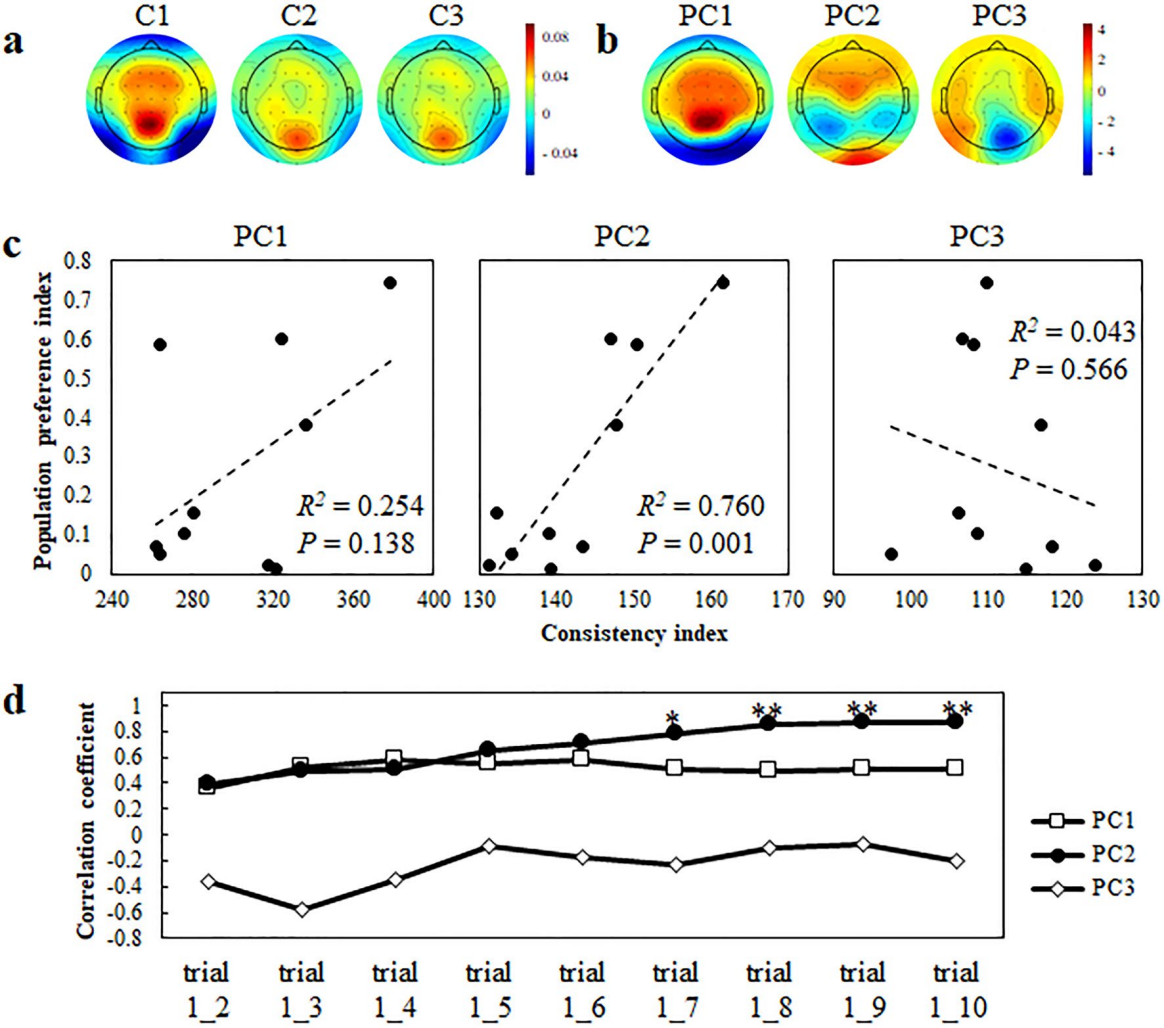
The relationship between PCA-based extraction of inter-trial consistency responses and population preferences. (**a**) Scalp topographies indicating canonical loadings (C1, C2, and C3) of the first three correlated components for the 3–80 Hz band when viewing the most popular TV commercial (i.e. the TV commercial with the highest population preference index in the stimuli dataset). The canonical loadings were computed for all paired 15-s (15,000 time points) trials and averaged across all participants, paired trials for each of the first three components. (**b**) Scalp topographies indicating the 1st, 2nd, and 3rd principal-component scores (PC1, PC2, and PC3, respectively) obtained from concatenated canonical loadings from all participants, paired trials and all of the first three components for the 3–80 Hz band when viewing the most popular TV commercial in the dataset. (**c**) Relationships between the population preference indices for ten TV commercials collected from a large audience and consistency indices representing the IP between the template PC score for the most popular TV commercial in the dataset and PC scores obtained similarly for each video. Dashed lines indicate the linear regressions between the indices. (**d**) Pearson correlation coefficients between the consistency indices and the population preference indices as a function of trial-pair datasets. The consistency index was computed using all possible trial pairs within the indicated trial number range. Bonferroni corrected, one-sided: *P < 0.05, **P < 0.01.

Figure 1a shows the topographical presentations of group-averaged canonical loadings (C1, C2 and C3), which are the correlation coefficients between projected canonical variates and multichannel EEG signals, when participants were viewing the TV commercial that showed the highest population preference index in the dataset (i.e. the most popular one). Since canonical variates are linear combinations of EEG signals with the eigenvector of the CCA as weight coefficients, canonical loadings indicate the contribution of each EEG signal to the canonical variates. The population preference index was calculated as the total number of unaided recalls divided by the number of times it was broadcast (see “Methods”). These topographic patterns were highly similar and symmetric, marked by occipital and parietal positivity and frontal and occipital-temporal negativity. The results, therefore, suggest that the simple CCA-based method does not separate distinct spatial patterns in canonical loadings across multiple components. Hereafter, to separate these spatial patterns, we added a principal-component analysis (PCA)-based method.

Figure 1b presents the 1st, 2nd, and 3rd principal-component scores (PC1, PC2, and PC3, respectively) obtained from canonical loadings of the first three correlated components (i.e. canonical variates) for the most popular TV commercial in the dataset. The three PC scores had distinct spatial distributions. Whereas the topographic pattern of PC1 closely resembled the first three correlation-maximised components, that of PC2 showed frontal and occipital positivity with temporal and parietal negativity, and PC3 was symmetric, with positivity over the temporal lobes and negativity over the medial parietal cortex. We hypothesised that these patterns would change in a population preference-dependent manner. To test this, we examined the association between the population preference index and consistency indices, which were computed as the inner product (IP) between the template PC scores for PC1, 2, and 3, which were obtained for the most popular TV commercial in the dataset, and the PC scores (PC1, 2, and 3) for each video. Regression analyses using PC1, 2, and 3 as independent variables showed that the consistency index calculated from PC2 independently predicted the population preference index, accounting for 76% of the variance [*F*(1,8) = 25.28, two-sided] (Fig. 1c). The predicted population preference index *y* was regressed as *y* = − 3.45 + 0.026*x*, where *x* is the consistency index calculated from PC2. The consistency indices calculated from PC1 [*F*(1, 8) = 2.71, two-sided] and PC3 [*F*(1, 8) = 0.36, two-sided] were not predictors of the population preference index (Fig. 1c). Although the group-averaged preference rating was also a predictor of the population preference index [*F*(1, 8) = 5.95, *R*^2^ = 0.43, *P* = 0.041, two-sided] (Fig. S1), the predictive accuracy was lower than that of the regression model of the consistency index calculated from PC2. The Akaike information criterion (AIC) for the regression model using the consistency index for PC2 as an independent variable showed a lower value (− 36.77) than the AIC for the model using group-averaged preference ratings (− 28.08). Since it is known that a difference in AIC of more than 10 (and 4) between two models indicates strong (and considerable) evidence for the selection of the model which showed lower AIC^15,16^, the results suggest that using brain data (i.e. PC2) obtained from a small group is a better choice than using group-averaged preference ratings of the small group. In addition, we confirmed that a stepwise multiple regression analysis using group-averaged preference ratings, PC1, 2, and 3 as independent variables indicated that the consistency index calculated from PC2 solely predicted the population preference index, accounting for 76% of the variance [*F*(1,8) = 25.28, two-sided]. The results were also confirmed by a statistically higher correlation coefficient between the population preference index and the consistency index (0.87) than the correlation coefficient between the population preference index and the group-averaged preference rating (0.65) (*t* = 2.402, *p* < 0.05). On the other hand, the participants’ recognition rate was not a predictor of the population preference index [*F*(1, 8) = 1.55, two-sided] (Fig. S2).

We then examined how the number of repetitive trials influences the consistency index of ten TV commercials by using Pearson’s correlation coefficients between the consistency indices and the population preference index. Figure 1d demonstrates the effects of the number of trials (*N*) on the correlation coefficients between the consistency index of TV commercials computed using from the 1st to *N*-th trials (_*N*_C_2_ trial pairs) and the corresponding population preference index. The correlation coefficients between the consistency indices calculated from PC2 and population preference indices increased as the number of trial pairs increased, such that the correlations were statistically significant with PC2 computed from canonical loadings obtained through seven trials (*r* = 0.78, *P* = 0.0036, Bonferroni corrected, one-sided). By contrast, the consistency indices calculated from PC1 and PC3 for all ten trial-pair datasets were not significantly correlated with the population preference index. These results indicated that multiple repetitive trials (in this case, seven trials) were needed to observe the correlation between the consistency indices calculated from PC2 and population preference indices.

### Frequency characteristics of the inter-trial consistency responses

**Figure 2:**
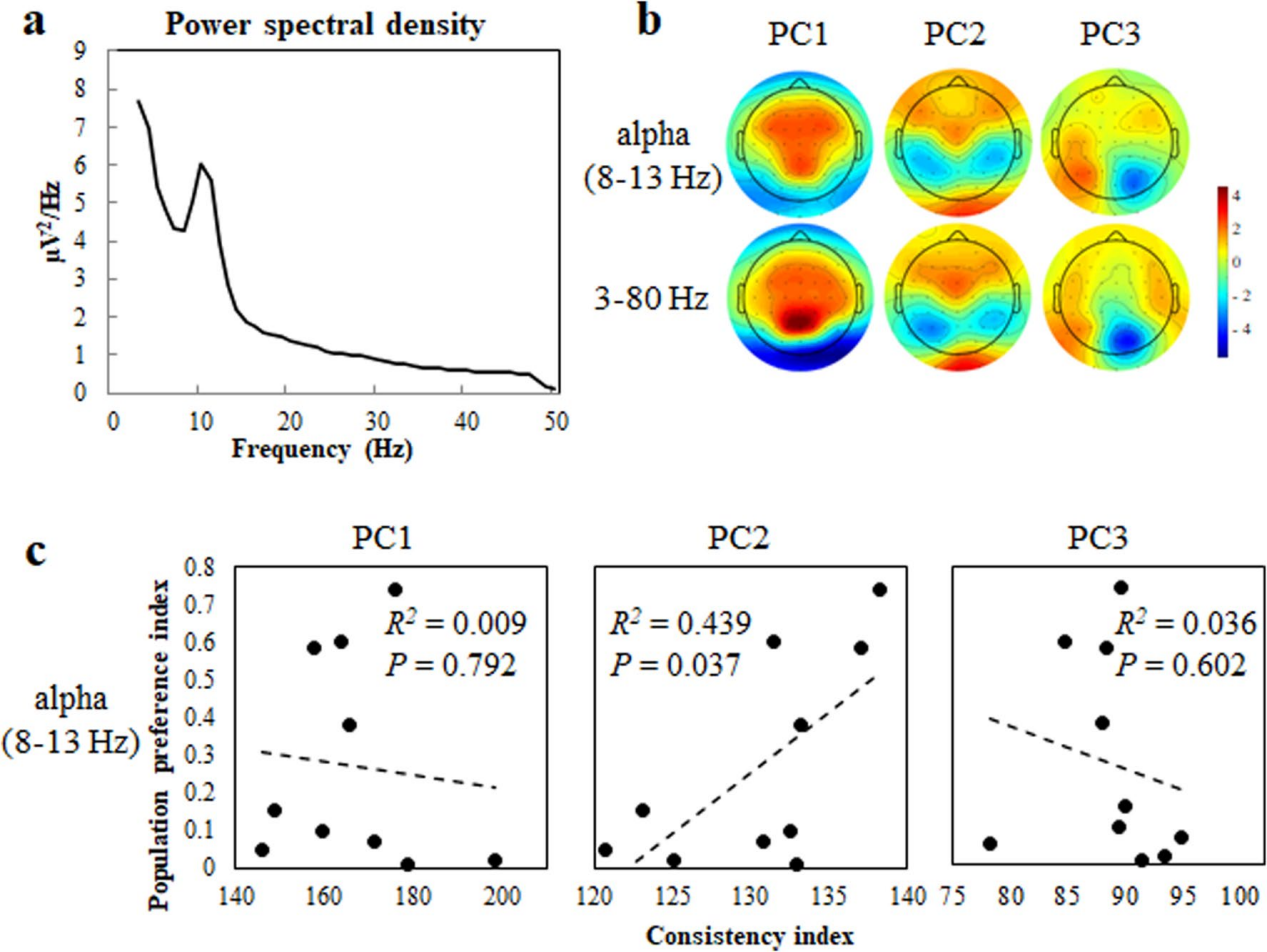
Neural activity patterns in the alpha band and population preferences. (**a**) Power spectral density of canonical variates averaged for the first three canonical variates, all TV commercials, and all participants. A characteristic peak is evident in the alpha band (~ 10 Hz). (**b**) Scalp topographies indicating PC1, PC2, and PC3 from canonical loadings of the first three correlated canonical components of alpha (top) and 3–80 Hz (bottom) bands while viewing the most popular TV commercial in the dataset. (**c**) Relationships between the population preference indices (as described for Fig. 1c) and consistency indices in the alpha band. Dashed lines indicate the linear regressions between the indices.

We next investigated the frequency characteristics of the canonical variates. Figure 2a shows the average power spectral density of the first three canonical variates, all TV commercials, and all participants. As a peak was present at ~ 10 Hz, characteristic of the alpha band, we analysed the consistency indices calculated from the PC of the alpha band (8–13 Hz). Similar topographic patterns were observed for PC scores from canonical loadings of the first three canonical variates of the alpha band and those at 3–80 Hz for the most popular TV commercial in the dataset (Fig. 2b). The consistency indices between the PC score for the most popular video and PC scores for the alpha bands were calculated to examine their association with the population preference index. A regression analysis showed that only the consistency index calculated from PC2 for the alpha band independently predicted the population preference index, accounting for 44% of the variance [*F*(1, 8) = 0.075, 6.27 and 0.29, two-sided for PC1, PC2, and PC3, respectively] (Fig. 2c). For these analyses, the predicted population preference index *y* was regressed as *y* = − 3.90 + 0.032*x*, where *x* is the consistency index calculated from PC2 for the alpha band. These results suggest that the specific spatial scalp distribution of alpha-band PC scores, that is, frontal and occipital positivity with temporal and parietal negativity, is related to the predictability of population preferences. However, the consistency index calculated from PC2 for 3–80 Hz band-pass-filtered data showed higher prediction accuracy of the population preference index than that for the alpha band (compare PC2 for Figs. 1c and 2c). Therefore, we used the consistency index for 3–80 Hz band-pass-filtered data for PC2 for subsequent analyses.

### A predictive model for population preferences

**Figure 3:**
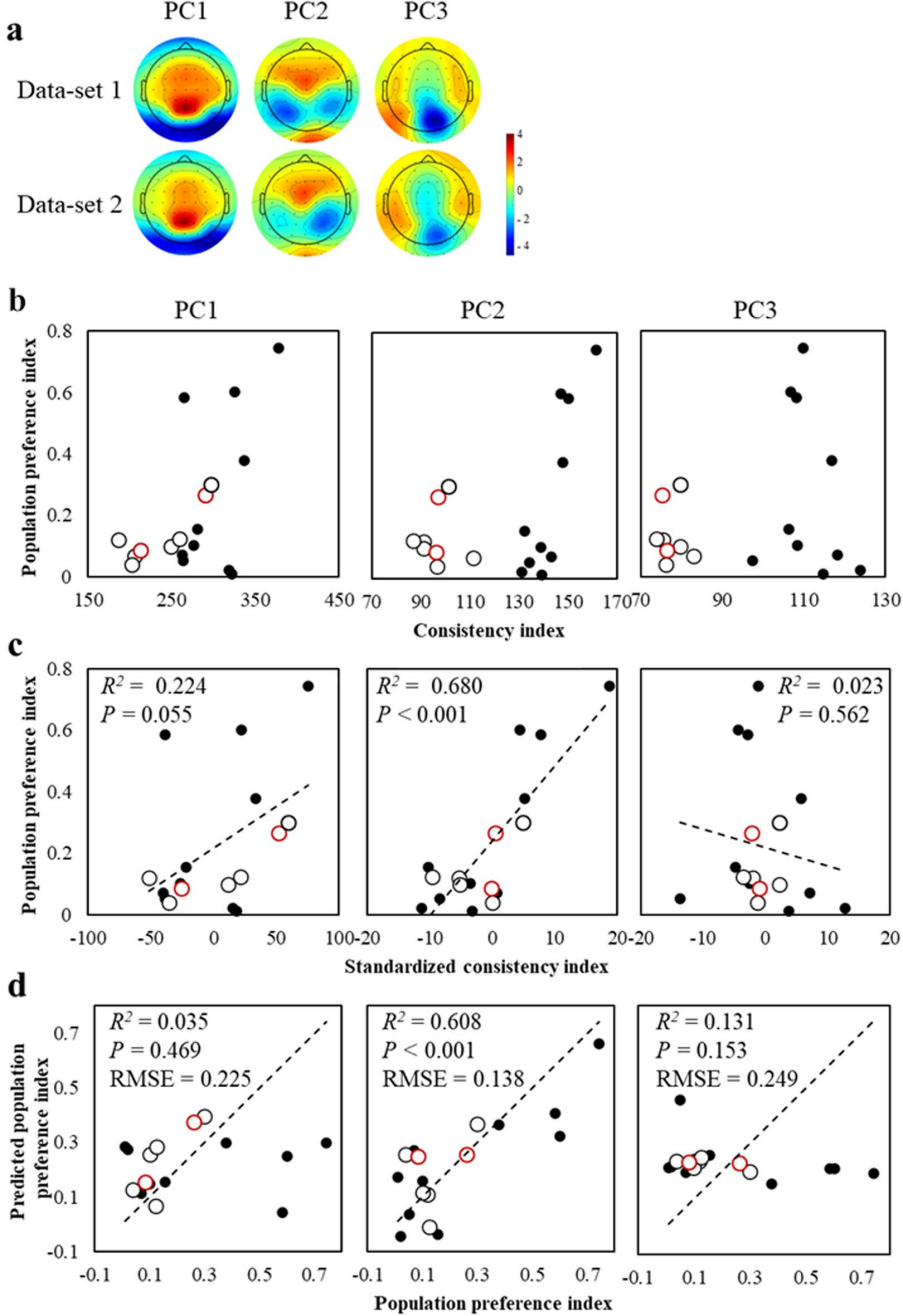
A predictive model adapted for two different datasets. (**a**) Scalp topographies for PC1, PC2, and PC3 obtained from canonical loadings of the first three canonical variates from different datasets show similar patterns. Dataset 1 represents data for ten TV commercials viewed by 23 participants (same as in Figs. 1 and 2); dataset 2 represents data for eight TV commercials viewed by 16 participants. (**b**) Population preference indices for 18 TV commercials collected from a large audience and consistency indices from dataset 1 (closed circles) and dataset 2 (open circles). (**c**) Same as for panel b, but mean values across stimuli were subtracted from the consistency index to control for the different IP distributions obtained from two different datasets. A model with the consistency index of the PC2 variable revealed a high Cook’s distance value (1.16) for one of eight TV commercials in dataset 2, which was removed from the consistency indices of PC1, PC2, and PC3; the population preference indices and consistency indices for the remaining 17 TV commercials were remodelled. Dashed lines indicate the linear regressions. (**d**) Observed-against-predicted population preference indices using the LOOCV method. Predicted population preference indices are for 17 TV commercials from regression models between the consistency and population preference indices versus the observed population preference indices from a large audience. Datasets 1 and 2 are shown as closed circles and open circles, respectively. Dashed lines indicate a perfect fit between observed and predicted data. RMSE, root mean square error. Red circles indicate data for two TV commercials (CM #16 and #17) that had not been broadcast when the EEG experiments were conducted. Population preference data for the two TV commercials were obtained after the experiments and submitted to LOOCV.

Given the strong correlation between the consistency index calculated from PC2 and the population preference index, we sought to validate the results on a different stimulus set, repeating the experiment with eight 15 s TV commercials and 16 different participants (dataset 2). Figure 3a shows that the topographies of PC scores obtained from canonical loadings of the first three canonical variates averaged across all stimuli were similar to those from the first dataset. We then calculated the consistency indices between the PC score for the most popular TV commercial and PC scores for the other TV commercials for each dataset, resulting in different distributions for the two different datasets (dataset 1 (closed circles) and dataset 2 (open circles)) (Fig. 3b and Fig. S3). We speculated that this was a result of the differences in the numbers of TV commercials and participants in the two datasets that were used for the PCA-based extraction. To remove the biases from the two datasets, we subtracted the mean value of the consistency index from each dataset, yielding normalised consistency indices, and performed regression analyses with the population preference index. The normalised consistency index calculated from PC2 provided a high Cook’s distance value (1.16), which is an estimate of the influence of a data point to find outliers^17^, for one of the eight TV commercials in dataset 2, suggesting that the data sample is an outlier. Therefore, this one sample was removed from the normalised consistency indices calculated from PC1, PC2, and PC3, and the population preference indices and normalised consistency indices for the remaining 17 TV commercials (ten from dataset 1 and seven from dataset 2) were remodelled. The subsequent regression analyses showed that the normalised consistency index calculated from PC2 independently predicted the population preference index, accounting for 68% of the variance [*F*(1, 15) = 31.91, two-sided] (Fig. 3c). The predicted population preference index *y* was regressed as *y* = 0.24 + 0.024*x*, where *x* is the normalised consistency index calculated from PC2. Again, values for PC1 and PC3 [*F*(1, 15) = 4.32 and 0.35, two-sided, respectively) were not significant predictors. We then evaluated the regression model using a leave-one-out cross-validation (LOOCV), which demonstrated good performance of the regression model using the normalised consistency index calculated from PC2 [*F*(1, 15) = 23.29, two-sided] (Fig. 3d). These data demonstrate that population preferences can be predicted from a dataset composed of multiple subdatasets with various numbers of participants and video stimuli. Note that population preference data for two TV commercials were obtained after the EEG experiments because they had not been broadcast when the EEG experiments were conducted (red circles in Fig. 3).

### The relationship between the spatiotemporal complexity of video stimuli and inter-trial consistency

**Figure 4:**
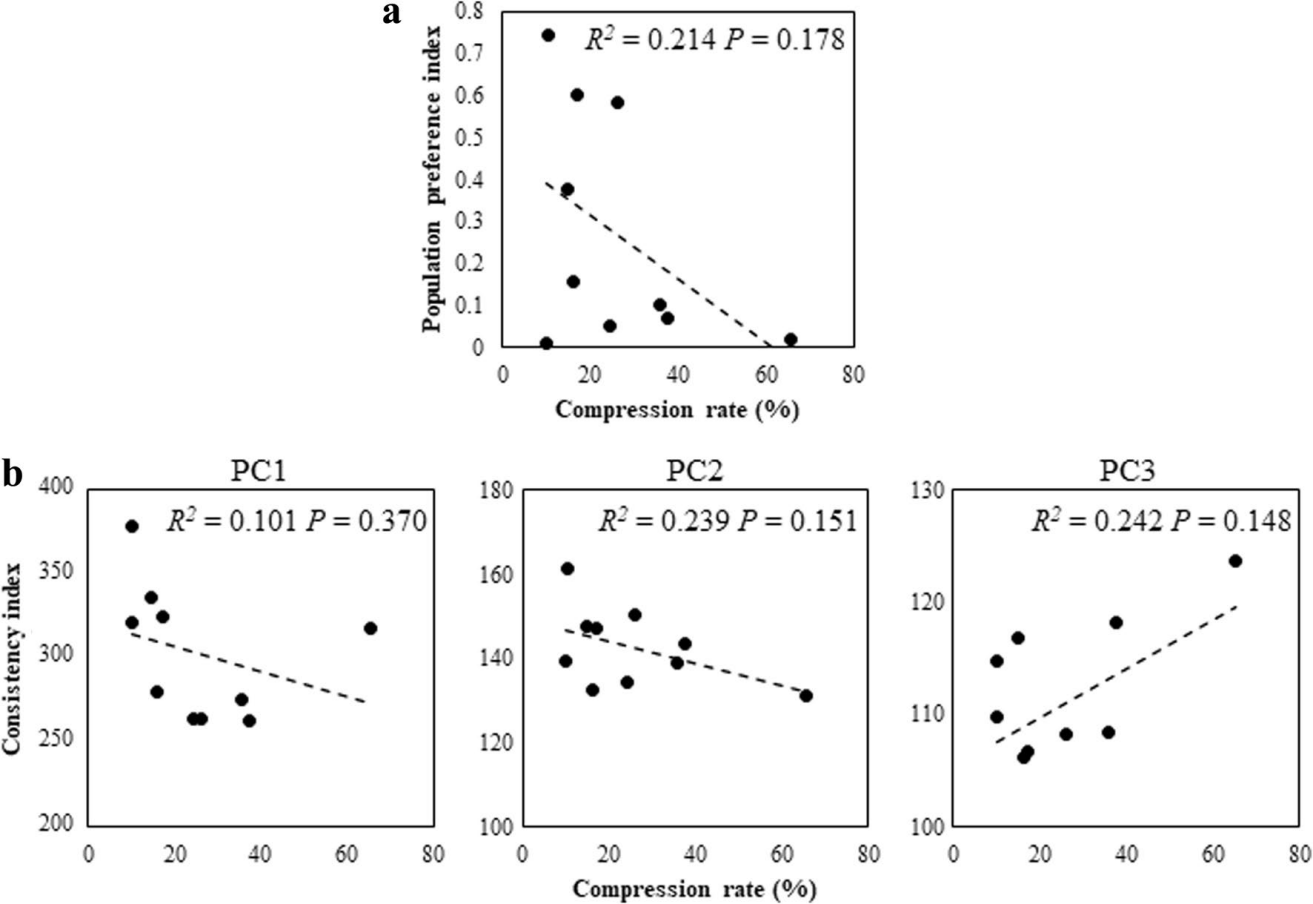
The relationship between the Lempel–Ziv compression rate of TV commercials and population preference. (**a**) Population preference indices (as described for Fig. 1c) and compression rates of TV commercials computed by the Lempel–Ziv data compression algorithm. Dashed line indicates the linear regression. (**b**) Consistency indices and compression rates as described for panel (**a**). Dashed lines indicate the linear regressions.

We found that the topographic patterns of PC scores with positivity over the occipital lobes were related to the predictabilities of the population preferences (see Figs. 1b and 2b). Since the topographic pattern suggested the contribution of the visual cortex, we speculated that the physical features of visual stimuli affect the consistency of EEG responses. Since it is known that the complexity of visual stimuli promotes attentional engagement^18^, visual and auditory cortices are shown to represent the complexity of naturalistic sensory stimuli^19^ and the complexity of movie clips can facilitate perceptual sensitivity^20^, we analysed if that the spatiotemporal complexity of the video stimuli contributed to the EEG consistency and the association between the consistency and the population preference. To estimate how much of the consistency was driven by the spatiotemporal complexity of the video stimuli, we examined the relationship between the consistency indices, population preference indices, and spatiotemporal complexity of TV commercials as computed as the compression rate by the Lempel–Ziv data compression algorithm (see “Methods”), where a higher compression rate indicates lower complexity of a video. A regression analysis revealed that the spatiotemporal complexity assessed as the compression rate of a TV commercial is not a predictor of the population preference index [*F*(1, 8) = 2.18, two-sided] (Fig. 4a). An additional stepwise regression analysis with the consistency indices calculated from PC1, 2, 3, and the compression rate showed that only PC2 independently predicted the population preference index, accounting for 76% of the variance [*F*(1,8) = 25.28, two-sided]. Similarly, the compression rate was not a predictor of the consistency indices calculated from PC1, PC2, and PC3 [*F*(1, 8) = 0.90, 2.51 and 2.56, two-sided, respectively) (Fig. 4b). These results suggest that the consistency in neural responses is not determined by spatiotemporal complexities of the stimulus, which can potentially induce prominent brain responses.

## Discussion

Here, we show that the consistency in neural responses to a repeatedly presented video stimulus, measured as spatiotemporal reproducibility of neural responses in a small sample of viewers, predicts population preferences. These data suggest that population preferences are associated with these spatiotemporal patterns in individual brain activity maintained after repeated presentations of a stimulus. Thus, these spatiotemporal patterns in brain activity obtained from a small sample are universal, presumably reflecting common brain activity patterns in a large population. As the population preference in the present study was defined as the TV commercial that a large population recalled as their favourite, these universal spatiotemporal patterns in brain activity observed in a smaller sample of viewers should be related to engagement and memory. Therefore, by measuring the consistency of neural responses across repetitive views among a small sample of individuals, we succeeded in extracting universal spatiotemporal activity patterns associated with population preferences.

Our proposed method revealed that by calculating a consistency index between the template PC2 score obtained for the TV commercial with the highest population preference index and PC2 scores for other TV commercials, we can accurately predict population preferences. By contrast, prior EEG studies have shown that intra-individual correlations assessed as the sum of the first three canonical correlation coefficients when participants viewed short video clips twice are associated with the engagement of viewers^2,5^. We think that our combined PCA-CCA method, utilizing 10 repetitive trials, is a different approach, which distinguishes spatial projection in the canonical loadings of the first three canonical variates, enabling us to detect the specified spatiotemporal pattern of neural responses linked to the popularity of a video stimulus. In our proposed method, we need to have EEG data for a TV commercial with a high population preference index, which is also different from the prior study showing that the inter-individual correlation of EEG activity was associated with population preferences^14^. Practically, it is possible to have such a dataset, however, it will be difficult to predict population preferences for more preferred TV commercials than the one on hand. It can be a potential disadvantage of our proposed method.

Intriguingly, it should be noted that group-averaged preferences showed lower performance in predicting population preferences compared to the consistency index obtained from the brain responses of the small group of people. Moreover, the participants’ recognition rate was not a good predictor of the population preference index. The results suggest that the brain responses obtained from a small group of people can tell more about population preferences than preference ratings or the recognition rate obtained from the small group.

Several works have shown that inter-individual correlation is associated with population responses^13,14^. Although these results were validated with the same set of participants and a new stimulus^14^, we utilised two separate datasets with different numbers of participants and stimuli, demonstrating the generalizability of the consistency index as a predictor of population preference. Therefore, we believe that we have provided the first evidence that population preference can be robustly predicted by a model with high generalisation ability. It should also be noted that our dataset includes two TV commercials that had not been broadcast when the EEG experiments were conducted. Therefore, their prediction using LOOCV (red circles in Fig. 3d) is similar to the situation in neuromarketing, where population preference has to be predicted before broadcasting a TV commercial.

An analysis of the power spectral density of the canonical variates revealed a peak at ~ 10 Hz, which is characteristic of the alpha band, consistent with the results from a previous study^14^. Furthermore, the consistency index calculated from PC2 of alpha band neural responses predicted the population preference, indicating a relationship between alpha-band activity and population preference. The spatiotemporal pattern of this activity, as well as that of a wider frequency range in neural responses, in PC2 indicated that the consistency is increased in the frontal and occipital cortices. This is consistent with a recent study showing that the prominent signals in response to salient stimuli (i.e. noisy flickering checkerboard stimuli) were from occipital electrodes placed over the lower visual cortex^8^. Although higher alpha power is known to be related to the lower arousal level^21^, it is hard to explain how such non-specific activity shows consistency across trials associated with population preferences. Further studies are needed to investigate the functional role of alpha consistency.

The spatiotemporal pattern in brain activity related to the consistency and preference indices should be also associated with memory because we measured population preference as an index of the number of unaided recalls of a “favourite” TV commercial. As for the relationship between preference and memory, a prior study showed that recognition memory of objects was enhanced by the simultaneous presentation of physically attractive celebrities^22^. Furthermore, stimuli (brand logos) that were recognised by participants were more likely to be preferred than those that were not recognised. Greater recognition confidence was significantly associated with an increased likelihood of preferring a stimulus^23^. Thus, recognition memory is intimately connected with preference. Good cognitive and memory performances are related to a tonic increase in alpha oscillations^24^, and alpha amplitudes in the parietal area increase when visual working memory is active^25^. Furthermore, subjective preferences positively correlate with the effective duration of the autocorrelation function of alpha waves measured in visual cortex^26^. In line with these studies, our findings suggest that the spatiotemporal pattern related to the consistency in the alpha band can be used as a biomarker to predict population responses involving both preference and memory.

Interestingly, we found that the complexity of the TV commercial, i.e. the calculated compression rate of the visual stimulus, did not predict population preference or consistency indices. These results indicate that neural consistency rather than the physical characteristics of the stimulus is the factor important for predicting population preference. This contrasts with a previous work that showed a significant correlation between the inter-individual correlation and the averaged luminance difference of stimuli^5^. However, the compression rate and average luminance difference are very different measures. Also, there were some other features, such as narrative, music, sound, and characters which were not evaluated in terms of complexity. It should be noted that the estimation of the complexity of multimodal components is technically difficult. Therefore, in future studies, we will investigate the relationship between multimodal stimulus characteristics and population responses.

In our experimental paradigm, we asked participants to fixate on a central cross and refrain from eyeblinks and eye movements during the video presentation since eyeblinks and eye movements contaminate EEG signals. A recent study, however, reported that naturalistic viewing conditions allowing participants’ eyeblinks and eye movements could enhance task engagement in an aesthetic rating task with a minimal impact on the quality of EEG signals^6^. We, therefore, think that a more liberal naturalistic viewing condition can be a better paradigm enhancing the engagement and memory of viewers in our future studies.

In summary, we demonstrated that the within-individual consistency in spatiotemporal patterns of brain activity responses to repetitive stimuli represents a potential biomarker for predicting the preference behaviours of a population. Thus, this method would be a beneficial tool for predicting population responses to videos and may become increasingly important in the future.

## Methods

### Participants and stimuli

For dataset 1, 32 healthy participants (14 female and 18 male; age range, 20–54 years; mean age, 25.2 years; standard deviation, 7.5 years) viewed ten 15 s TV commercials aired during 2016. For dataset 2, 22 healthy participants (11 female and 11 male; age range, 27–48 years; mean, 40.0 years; standard deviation, 7.2 years) viewed eight 15 s TV commercials aired during 2017. These stimuli were presented on a 24-inch LCD monitor (BenQ XL2420; BenQ Corporation, Taiwan) at a refresh rate of 60 Hz using NBS Presentation (Neurobehavioral Systems Inc., Berkeley, CA, USA). All participants provided written informed consent. The study was approved by the RIKEN ethics committee and conducted in accordance with the Declaration of Helsinki. None of the participants had any history of neurological disease.

Participants were seated 100 cm from the LCD monitor with their head positions maintained with a chin rest. The size of each video was 720 × 480 pixels (19.5 × 13 cm), with a visual angle of 11.1° × 7.4°. Sound was played back with computer speakers adjusted to a comfortable listening volume. Participants were instructed to neutrally view the video stimuli and were asked to fixate on a cross symbol (size, 0.6 × 0.6 cm; visual angle, 0.3° × 0.3°) overlaid on the video stimulus. Participants were asked to refrain from eyeblinks and eye movements during the video presentation since eyeblinks and eye movements contaminate EEG signals. Participants were presented with each TV commercial ten times in a randomised order.

After the experiment, participants were required to rate each of the TV commercials [on a scale rating from 1 (extremely dislike) to 5 (extremely like)]. Participants were also required to answer a questionnaire regarding their familiarity with TV commercials after participating in the EEG experiment. Specifically, participants answered if they knew each TV commercial. The participants’ recognition rate for each TV commercial was computed as the total frequency count of “knew” divided by the number of participants.

The 18 TV commercials were selected among TV commercials for products or services with a wide range of population preference indices (obtained from CM Soken Consulting, TOKYO KIKAKU Co., Ltd., Japan; see the details below) (Table 1). Since two TV commercials (CM #16 and # 17) had not been broadcast when the EEG experiments were conducted, population preference data for the two TV commercials were obtained after the experiments. These TV commercials were broadcast in Japan from 2016 to 2017.

**Table 1:** TV commercials and population preference data.

No	Product/service advertised in TV commercial	Population preference index	Data set	Period of a broadcast (month)	Consumer sample size
1	Canned coffee	0.58	1	4	12,000
2	Canned coffee	0.07	1	4	12,000
3	Cup noodle	0.38	1	4	12,000
4	Cup noodle	0.10	1	7	21,000
5	Mobile carrier	0.74	1	2	6000
6	Mobile carrier	0.01	1	2	6000
7	Beer	0.60	1	4	12,000
8	Beer	0.05	1	8	24,000
9	Job board	0.15	1	1	3000
10	Job board	0.02	1	27	81,000
11	Mobile carrier	0.12	2	4	12,000
12	Face wash	0.07	2	7	21,000
13	Energy drink	0.04	2	11	33,000
14	Frozen food	0.30	2	10	30,000
15	Beer	0.26	2	3	9000
16	Beer	0.08	2	3	9000
17	Beer	0.12	2	9	27,000
18	Beer	0.10	2	2	6000

### Population preference data

The population preference index of all TV commercials used in this study were obtained from CM Soken Consulting service run by TOKYO KIKAKU Co., Ltd (https://www.cmdb.jp/cmlikability/). These scores are assessed from the total numbers of unaided recalls, obtained as top three favorite TV commercials from each consumer, for a favourite TV commercial gathered from 3,000 consumers every month (female and male; age range, from 6 years old to over 60 years old) (Table 1). To remove the biases caused by the number of times a commercial was broadcast, the population preference index was calculated as the total number of unaided recall divided by the number of times the commercial was broadcast.

### EEG recordings

While the participants viewed the video stimuli, high-density scalp EEG signals were recorded with an amplifier (BrainAmp MR plus; BrainProducts GmbH, Gilching, Germany) using a cap with 63-channel (ch) Ag/AgCl electrodes located on the basis of the international 10/10 system (EASYCAP; EASYCAP GmbH, Herrsching, Germany), with a ground electrode at AFz and a left earlobe reference electrode. EEG signals were recorded at a sampling rate of 1000 Hz by recording software (BrainVision Recorder; BrainProducts GmbH, Gilching, Germany) and were rereferenced offline to the average from the left and right earlobes. Online lower and higher cutoff frequencies of the EEG amplifier were set to 0.016 Hz and 250 Hz, respectively. Horizontal and vertical electrooculographic signals were also recorded. Electrode impedance was kept below 10 kΩ.

### EEG preprocessing

EEG epochs, including the onset and offset of the video stimulus, were first extracted from the continuous EEG/electrooculographic data. This constructs 19,000 ms-long epochs, which included EEG data from 2000 ms prestimulus and 2000 ms poststimulus.

An offline band-pass 3–80 Hz finite impulse response filter was applied to the epochs. Next, an independent component analysis-based automatic artefact removal procedure [automatic EEG artefact detector based on the joint use of spatial and temporal features (ADJUST)] was applied to remove generic artefacts, eye movements and eye blink-related artefacts^27^. We excluded participants with more than 0.001% of data points exceeding ± 100 uV criteria for further analyses. As a result, 23 participants for dataset 1 and 16 participants for dataset 2 survived for further analysis. Current source density transformation based on the spherical spline surface Laplacian method was then applied to minimise the influence of volume conduction^28,29^. Then, an offline band-pass finite impulse response filter was applied to the EEG epochs at one of the following frequency ranges: 8–13 Hz (alpha), or 3–80 Hz. Finally, a 50 Hz notch filter was applied to reduce the effects of power line noise.

All offline data processing was performed using in-house-made MATLAB (MathWorks, Natick, MA, USA) scripts, EEGLAB^30^ and ADJUST^27^.

### EEG inter-trial consistency

To assess the degree of consistency of brain responses across EEG trials, a CCA-based method was applied between the trial-pairwise EEG trials across repetitive ten trial data matrices (***X***_1_,…, ***X***_10_) in a round-robin design in which there are _10_C_9_ = 45 unique trial pairs as follows: trial pair (*n*, *m*) = [(1, 2), (1, 3),…, (9, 10)], for each video stimulus within each individual. CCA is a conventional statistical method for extracting the linear combinations of data variables that give maximal correlations between pairwise datasets^31^. CCA is useful for detecting correlated components between complex time series data from a pair of dynamical systems^32^. Specifically, our CCA-based method finds the eigenvectors (***a*** and ***b***) as weight coefficients, which maximises the correlation coefficient, calculated as

$$\rho \left(\varvec{u,v}\right)=\frac{{\text{Cov}}[\varvec{u, v}]}{\sqrt{{\text{Var}}[\varvec{u}]}\sqrt{{\text{Var}}[\varvec{v}]}} ,$$

between the canonical variates ***u*** and ***v*** composed of linear combinations of 63-ch EEG signals (***X***_*n*_ and ***X***_*m*_; 63 × 15,000 data points) as follows:

$${\varvec{u}}={\varvec{a}}^{\text{T}}{\varvec{X}}_{n},$$

$${\varvec{v}}={\varvec{b}}^{\text{T}}{\varvec{X}}_{m},$$

wher﻿e ^T^ denotes the matrix transpose. To investigate topographical patterns and the extent to which EEG signals contribute to the canonical variates, the canonical loading vector, which is composed of a set of correlation coefficients between projected canonical variates and EEG signals, was assessed. Note that eigenvectors and canonical loadings are not unique in terms of the sign. To align the signs of canonical loadings, we counted the number of positive signs of 63 elements that composed the canonical loading vector and flipped the signs of the canonical loading if the number of positive signs was less than 32.

Note that this sign reversal procedure was not necessary because we conducted PCA of canonical loadings subsequently. We took this procedure to visualize the averaged canonical loadings patterns. Figure 1a shows the canonical loadings averaged across trial pairs and participants for each of the first three canonical variates (Fig. 1a).

### Assessment of consistency index

The simple CCA-based method does not separate distinct spatial patterns in canonical loadings across multiple components (Fig. 1a). Therefore, PCA was employed to separate spatial projections in the canonical loadings, consisting of 63 channels and *N* samples per stimulus, where *N* represents a concatenation of data from the participants, the trial pairs and the first three components. Specifically, the formats of the datasets were as follows: dataset 1, 63 ch × 10 stimuli × 6210 samples {23 participants × 45 trial pairs × 6 components [3 (first three components) × 2 (***u*** and ***v***, obtained from data ***X***_*n*_ and ***X***_*m*_, respectively)]}; dataset 2, 63 ch × 8 stimuli × 4320 samples [16 participants × 45 trial pairs × 6 components]. PCA was conducted so that PC coefficients, which are the elements of eigenvector in PCA as in the case of CCA, were obtained with their size corresponding to the length of the dataset (i.e. 62,100 for dataset 1, 34,560 for dataset 2). Although canonical loadings data are not unique regarding the sign, this PCA approach resolves the issue by aligning the signs of canonical loadings with similar spatial patterns. The consistency index was computed by the following two steps. Step 1, the first three principal-component scores (PC scores) were extracted by conducting PCA of the canonical loadings data from dataset 1 and dataset 2, and the topographical patterns of the PC scores were computed from PC coefficients and canonical loadings for each TV commercial. Since PC scores are not unique in terms of the sign, we counted the number of positive signs of 63 elements and flipped the signs of them if the number of positive signs was less than 32. Step 2, the consistency index was calculated as the IP between the PC score obtained for the most popular TV commercial (see “Population preference data”) among all TV commercials and other PC scores obtained for other TV commercials.

### Power spectral density analyses

The canonical variates were transformed to the frequency domain using a fast Fourier transform, yielding an EEG power spectral density ranging from 1 to 50 Hz. The power spectral densities were then averaged across trial pairs, participants, TV commercials and the first three components.

### Assessment of video complexity

To evaluate the complexity of the video stimuli, their compression rates were calculated. First, the RGB information of each pixel at every frame was extracted from avi format video files for each TV commercial. Next, the Lempel–Ziv complexity^33^ of each pixel across every frame (720 × 480 pixels × 3 RGB × 30 fps × 14 s) was assessed using the MATLAB code “calc_lz_complexity.m”^34^. The compression rate was calculated as the file size of pre-Lempel–Ziv complexity divided by that of post-Lempel–Ziv complexity.

### Statistical analyses

Regression analyses between the consistency indices and population preference indices were conducted using IBM SPSS statistics software (version 25).

The LOOCV approach was used to assess the accuracy of the regression model to predict population preference index, and the correlation coefficient and root mean square error were estimated. This LOOCV approach consists of training a model with the complete dataset except for one point, and then that point is predicted by the model, enabling accuracy statistics to be estimated. The process is iterated 18 times, and the root mean square error is calculated. Regression analyses were conducted with IBM SPSS statistics software (version 25).

Regression analyses between compression rates and population preference indices or IPs were conducted with IBM SPSS statistics software (version 25). The statistical significance level was set to *p* = 0.05 for all statistical tests.

## Supplementary Information

Supplementary Figures.

## Data Availability

The data and code supporting the current study have not been deposited in a public repository because a nondisclosure agreement is required but are available from the corresponding author on request after the nondisclosure agreement.
