# Supplementary material for: Spatiotemporal consistency of neural responses to repeatedly presented video stimuli accounts for population preferences
Source: Sci Rep. 2023 Apr 4;13:5532. doi: 10.1038/s41598-023-31751-0 (PMC10073227; doi:10.1038/s41598-023-31751-0)
Supplement: Supplementary file 1 — Supplementary Figures. [file 41598_2023_31751_MOESM1_ESM.docx]

**Spatiotemporal consistency of neural responses to repeatedly presented video stimuli accounts for population preferences**

Ayaka Hoshi^1,2^, Yuya Hirayama^1^, Fumihiro Saito^1^, Tatsuji Ishiguro^2^, Hiromichi Suetani^1,3^, Keiichi Kitajo^1,4,5^*

^1^RIKEN Center for Brain Science, 2-1, Hirosawa, Wako, Saitama, 351-0198, Japan

^2^KIRIN Central Research Institute, Research & Development Division, Kirin Holdings Company, Limited, 26-1-12-12, Muraoka-Higashi 2-chome, Fujisawa, Kanagawa, 251-8555, Japan

^3^Faculty of Science and Technology, Oita University, 700, Dannoharu, Oita, 870-1192, Japan

^4^Division of Neural Dynamics, Department of System Neuroscience, National Institute for Physiological Sciences, National Institutes of Natural Sciences, 38 Nishigonaka, Myodaiji, Okazaki, Aichi, 444-8585, Japan

^5^Department of Physiological Sciences, School of Life Science, The Graduate University for Advanced Studies (SOKENDAI), 38 Nishigonaka, Myodaiji, Okazaki, 444-8585, Japan

*Correspondence: kkitajo@nips.ac.jp.


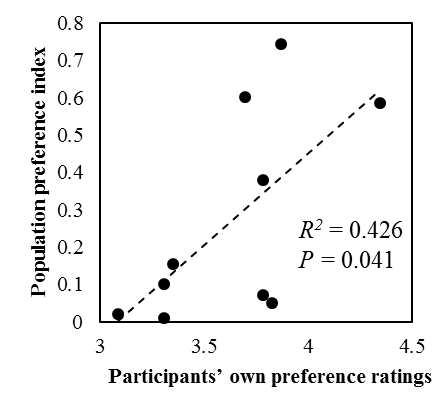
**Supplemental Information**

**Figure S1. Population preference can be predicted by participants’ preference ratings**

The relationship between the population preference indices for ten TV commercials collected from a large audience and the mean ratings assigned to each advertisement by the study participants (*N* = 23). Dashed lines indicate the linear regression between the indices.


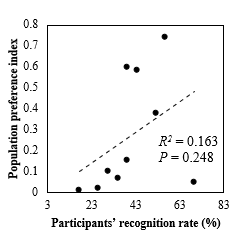
**Figure S2. Population preference cannot be predicted by participants’ recognition rate.**

The relationship between the population preference indices for ten TV commercials collected from a large audience and the recognition rate of each advertisement by the study participants (*N* = 23). Dashed lines indicate the linear regression between the indices.

**
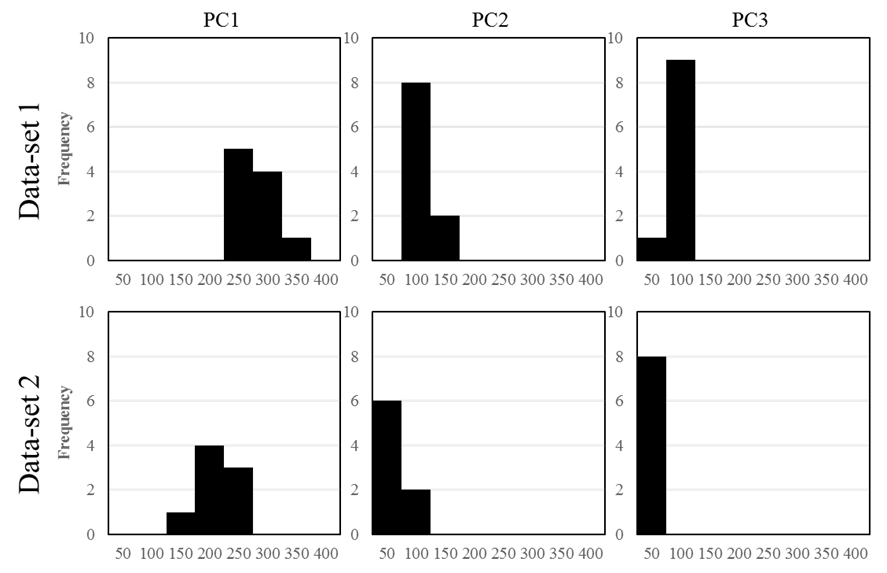
Figure S3. Two datasets have different IP histogram distributions.**

The consistency indices computed from the PC score for the most popular TV commercial and PC scores for the other TV commercials indicated different distributions for the two different datasets.
